# Drug screening of cancer cell lines and human primary tumors using droplet microfluidics

**DOI:** 10.1038/s41598-017-08831-z

**Published:** 2017-08-22

**Authors:** Ada Hang-Heng Wong, Haoran Li, Yanwei Jia, Pui-In Mak, Rui Paulo da Silva Martins, Yan Liu, Chi Man Vong, Hang Cheong Wong, Pak Kin Wong, Haitao Wang, Heng Sun, Chu-Xia Deng

**Affiliations:** 1 Cancer Centre, Faculty of Health Sciences, University of Macau, Macau, China; 2 State-Key Laboratory of Analog and Mixed-Signal VLSI (AMSV), University of Macau, Macau, China; 3 Department of Electrical and Computer Engineering, Faculty of Science and Technology, University of Macau, Macau, China; 4 Instituto Superior Técnico, Universidade de Lisboa, Lisboa, Portugal; 5 Department of Computer and Information Science, Faculty of Science and Technology, University of Macau, Macau, China; 6 Department of Electromechanical Engineering, Faculty of Science and Technology, University of Macau, Macau, China

**Keywords:** Chemotherapy, Chemotherapy

## Abstract

Precision Medicine in Oncology requires tailoring of therapeutic strategies to individual cancer patients. Due to the limited quantity of tumor samples, this proves to be difficult, especially for early stage cancer patients whose tumors are small. In this study, we exploited a 2.4 × 2.4 centimeters polydimethylsiloxane (PDMS) based microfluidic chip which employed droplet microfluidics to conduct drug screens against suspended and adherent cancer cell lines, as well as cells dissociated from primary tumor of human patients. Single cells were dispersed in aqueous droplets and imaged within 24 hours of drug treatment to assess cell viability by ethidium homodimer 1 staining. Our results showed that 5 conditions could be screened for every 80,000 cells in one channel on our chip under current circumstances. Additionally, screening conditions have been adapted to both suspended and adherent cancer cells, giving versatility to potentially all types of cancers. Hence, this study provides a powerful tool for rapid, low-input drug screening of primary cancers within 24 hours after tumor resection from cancer patients. This paves the way for further technological advancement to cutting down sample size and increasing drug screening throughput in advent to personalized cancer therapy.

## Introduction

Precision Medicine is the tailoring of clinical strategies based on diversification of genetic, behavioral and environmental backgrounds of individual patients^1^. In oncology, this involves the diagnosis, prognosis and design of therapeutic strategies for each patient, including but not restricted to chemotherapy, radiotherapy, tumor resection, and immunotherapy. Rational design of therapeutic strategies is critical to treating cancer. Cancer is one of the lethal diseases threatening millions of people worldwide, accounting for approximately 13% of all deaths globally^2^. The choice of therapy depends on the type and stage of cancer, past response rates, legal and clinical infrastructure, patient’s health conditions, *etc*. While radiotherapy is priority for brain tumors and nasopharyngeal cancers, chemotherapy is frequently used as standard protocol for many cancers, or as adjuvant therapy for surgically resected tumors. Although all clinically approved drugs and drug combinations have been tested *in vitro* using cultured cells, *in vivo* using animal models and in clinical trials, there is no guarantee of success for any case. Furthermore, due to insufficient knowledge of cancer etiology, diversity of cancer types and properties, relapse and metastasis, *etc*, cancer treatment tends to be ineffective or results in excessive side effects. In addition, societal issues like legal regulations of standard therapy, exorbitant treatment costs, *etc*, are additional burdens on cancer patients. Hence, there is a call for testing drug susceptibility of cancers at high reliability, low cost and less pain.

The major hurdle to drug screening on primary cancer samples is low sample input. Leukemia patients provide the most abundant source, where over 10 million cancer cells can easily be isolated from 2 mL patient blood. In contrast, mammary tumors of 2 × 2 cm may result in less than 1 million cells in sum after dissociation in the worst case. Sample quality also differs immensely depending on shipment time and storage method. In order to circumvent this issue, tumor amplification by two-dimensional (2D) monolayer cell culture^3–5^, three-dimensional (3D) organoid culture^6, 7^ and patient-derived xenograft (PDX) models^8, 9^ has been explored. Cultured circulating tumor cells (CTCs) from liquid biopsies^10, 11^ have also been tested. In spite of this advancement on the laboratory scale, the majority of hospitals worldwide do not possess adequate infrastructure and funding for implementation. Moreover, there are concerns of genetic and phenotypic fidelity of amplified tumors, although evidence of genetic stability especially in aggressive driver gene mutation(s)^12^ has been suggested. Consequently, development of drug screening methods on primary cancer samples remains indispensable.

To circumvent the sample input problem, drug screening platforms based on nano- and micro- volume fluidic channels have been vastly explored in the past decade^10, 11, 13–20^. The use of micro-volume channels not only reduces sample size, but also reduces cost as less reagents are consumed. In this study, we exploited a 2.4 × 2.4 cm polydimethylsiloxane (PDMS) based microfluidic chip, which employs droplet microfluidics to screen for drugs against cancer cell lines and cells dissociated from human primary tumors. PDMS has long been used in clinical applications due to its low cytotoxicity^21^, and its fabrication is relatively simple and cheap^22^. Eventually, our assay enabled screening of 5 conditions for every 80,000 cells under current circumstances, with a rapid turnover time of 24 h. In addition, screening conditions have been adapted to both suspended and adherent cancer cells, giving versatility to potentially all types of cancers. In comparison to published assays (Table 1), our assay has excellent performance based on its sample input and screen throughput. Moreover, given that input cell concentration and loading volume could be adjusted, there is much flexibility for the number of assay conditions to be screened on chip based on sample input size and throughput requirement. Furthermore, our readout method enables us to capture single cell drug response, thus providing the opportunity to observe and quantify differential drug response of single cells, without compromising population analysis using multiple droplets for each drug treatment condition. Hence, our assay provides a powerful tool for rapid, low-input drug screening of primary cancers.

**Table 1:** Current microfluidic technologies for drug screening.

Sample type	Subject	Total sampled conditions	Technology	Reference
Protein	Enzyme	704	Droplet microfluidics	13
Cell line	Cells in agarose	23 × 23 = 529	Array printing	14
	Cells	3328	Nano-well patterning	15
	Bacteria	20	Flow microfluidics	16
	Suspended and adherent cells	2 channels of 5 conditions	Droplet microfluidics	This study
Primary tumor	Leukemia cells	1266	Plate reader assay^#^	17
	Lung cancer and stromal cells	3	Flow microfluidics	18
	Multiple myeloma cells	1 drug × <5 dose	Flow microfluidics	19
	T2 breast tumor	16	Implanted chip	20
	Primary tumor dissociated cells	5–10 conditions depending on cell number	Droplet microfluidics	This study
Cultured tumor/CTC	Cultured CTC	38 × 6 = 228	*Ex vivo* culture	10
	Cultured single CTC	Dependent on cell amplification	Trap and release → *in vitro* culture	11

^#^This is not microfluidics-based assay.

## Materials and Methods

### Cancer cell lines and cell culture

Jurkat E6.1 cells (ATCC^®^ TIB-152™) and MDA-MB-231 cells (ATCC^®^ HTB-26™) were used as models for suspended and adherent cancer cell lines respectively. Jurkat cell line was derived from human acute T cell leukemia, whereas MDA-MB-231 cell line was derived from human metastatic breast adenocarcinoma.

Jurkat cells were cultured in Advanced RPMI 1640 medium (Life Technologies, USA) supplemented with 5% fetal bovine serum (FBS) (Gemini, USA), 100 U/mL Penicillin-Streptomycin (Life Technologies, USA), 2 mM L-glutamine (Life Technologies, USA), and 10 mM HEPES pH7.4 (Life Technologies, USA).

MDA-MB-231 cells were cultured in Dulbecco’s Modified Eagle Medium (Life Technologies, USA) supplemented with 5% FBS, 100 U/mL Penicillin-Streptomycin and 2 mM L-glutamine.

All cells were cultured in humidified incubator at 37 °C supplemented with 5% CO_2_.

### Primary tumor and tumor dissociation

All human studies were conducted with the approval of the Panel on Research Ethics of University of Macau and the Research Ethics Committee of Kiang Wu Hospital, according to the Materials Transfer Agreement between University of Macau and Kiang Wu Hospital. Informed consent for sampling and publication without identifiable information was obtained from all participating patients. All patient sample names were double encoded by the university and the hospital, respectively, to remove any trace of patient identity during sample collection, transfer, processing and analysis. Primary tumors were obtained from surgery conducted at Kiang Wu Hospital immediately after tumor resection. Tumor tissue was dissociated as previously described^23^. Briefly, tumor tissue was first cut into small pieces by a scalpel, then transferred to a 50 mL conical tube containing 5 mL Digestion Buffer I (DMEM/F12 medium containing 5% FBS, 5 μg/mL insulin, 500 ng/mL hydrocortisone, 10 ng/mL epidermal growth factor (EGF), 20 ng/mL cholera toxin, 300 U/mL collagenase III and 100 U/mL hyaluronidase), and digested for no more than 12 h with shaking at 100 rpm in humidified incubator at 37 °C supplemented with 5% CO_2_. After spinning down at 400 g at ambient temperature for 2 min, the cells were resuspended with 2 mL Digestion Buffer II (DMEM/F12 medium containing 5 mg/mL dispase II and 0.1 mg/mL deoxyribonuclease I), followed by digestion at ambient temperature for 5 min. The cells were then washed with 10 mL HBSS (Life Technologies, USA). 2 mL RBC lysis buffer (eBioscience, USA) was used to lyse red blood cells at ambient temperature for 3 min; this step was repeated until the solution became translucent. 12 mL HBSS (Life Technologies, USA) was finally added to stop the lysis. Dissociated cells were extracted by centrifugation of the filtrate through a 40 μm strainer (Falcon, USA). Lastly, the cells were resuspended in StemMACS iPS-Brew XF medium (Miltenyl Biotec, USA) and used for drug screening on chip.

### Microfluidic chip design and fabrication

A previously reported polydimethylsiloxane (PDMS)-based microfluidic device with a bypass channel around a droplet formation well^24^ was modified to enable robust droplet formation and storage in this study. A narrow restriction feature (15 μm × 150 μm) was put next to the droplet formation well (300 μm × 1150 μm) to facilitate droplet formation in the well during sample loading. A neck (100 μm × 225 μm) at the entrance of the droplet formation well was made to improve droplet formation, restrict crosstalk with subsequent flow during sample loading and prevent droplet escape during overnight incubation at 37 °C. Supplementary Figure 1 illustrated the chip design.

Soft photolithography by photomask (Shenzhen Newway, China) was used to fabricate SU-8 negative photoepoxy (Microchem, USA) on silicon wafer (Harbin Tebo Technology, China) following standard procedures to make the patterned wafers. The patterned wafers used in this study were determined to be 62–78 μm in height using KLA-Tencor AlphaStep D-600 Stylus Profiler (KLA-Tencor, USA). Polydimethylsiloxane (Dow Corning, USA) at 1:7 base to curing agent ratio (w/w) was poured onto the patterned wafers, baked in an oven at 65 °C for 25 min, and peeled off to generate PDMS slabs. Lastly, the PDMS slabs were plasma bound to 2.4 × 2.4 cm No. 1.5 square glass coverslips using Harrick Plasma PDG-002 Expanded Plasma Cleaner (Harrick Plasma, USA) to generate the ready-to-use microfluidic chips after baking at 65 °C overnight.

### On chip drug screening assay

All drugs used in this study were listed in Supplementary Table 1. Bortezomib and Vorinostat were chosen as target drugs for leukemia, i.e. Jurkat cells^25^, whereas Cisplatin and Epirubicin were chosen as target drugs for breast cancer, i.e. MDA-MB-231 cells^23, 26^. Another consideration of the chosen drugs was diverse therapeutic targets.

On chip drug screening was performed using the PDMS-based microfluidic chip as described above. A 500 μL glass syringe (Hamilton, USA) and polytetrafluoroethylene (PTFE) tubings with appropriate bore (Cole Parmer, USA) was used to connect between the syringe pump (Harvard Apparatus PHD Ultra Syringe Pump, USA) and the microfluidic chip. Fluorinert^®^ FC-40 (Sigma-Aldrich, USA) supplemented with 2% 008-Fluorosurfactant (Ran Biotechnologies, USA) was used as oil phase; relevant cell culture medium, supplemented with 1% (w/v) methyl cellulose (Sigma-Aldrich, USA) was used as aqueous phase. Cells treated with 0.1% dimethyl sulfoxide (DMSO) were used as negative control.

Briefly, cells at final concentrations of 1–2 × 10^6^ cells per mL were aliquoted in 0.2 mL PCR tubes, then mixed with corresponding drugs and 2 μM ethidium homodimer 1 (Life Technologies, USA) by manual pipetting. Next, 100–200 nL cell-drug mixtures were loaded into the tubing, consecutively segregated by oil phase at withdrawal rate of 200 μL/h by syringe pump. After loading all mixtures, the tubing was inserted into the microfluidic chip, which had been back-flushed with oil phase at 500 μL/h by syringe pump. After that, the mixtures were infused at 25 μL/h by syringe pump. Finally, the inlet and outlet tubings were cut and sealed with Vaseline (Vaseline, USA). The chips were placed in 150 mm cell culture dish (Corning, USA) containing wet paper towels, and transferred to humidified incubator at 37 °C supplemented with 5% CO_2_ for 16–24 h incubation. Brightfield and red fluorescence images (Ex. 531/40 nm, Em. 593/40 nm) were taken under 10x magnification (Life Technologies EVOS FL Imaging System, USA).

### Microtiter plate drug screening assay

Microtiter plate drug screening assays were carried out on 96-well clear round flat-bottom plates (Corning, USA) or 384-well white square flat-bottom plates (Corning, USA).

First, 5.0 × 10^5^ or 1.0 × 10^5^ cells were seeded per well for 96-well and 384-well plates respectively. Drugs were diluted with Dulbecco’s phosphate-buffered saline (DPBS) (Life Technologies, USA), and subsequently added to achieve final drug concentrations as indicated on the graphs. Afterwards, the plates were transferred to humidified incubator at 37 °C supplemented with 5% CO_2_ for 16–24 h incubation. Finally, Alamar Blue assay was used to measure cell viability^27^. Fluorescence intensity (Ex. 560 nm, Em. 590 nm) using auto-cutoff was measured from bottom on plate reader (Molecular Devices SpectraMax M5 Plate Reader, USA). Cells treated with 0.1% dimethyl sulfoxide (DMSO) were used as negative control, while no cells were added to blank control. All experiments were performed in triplicate for 96-well plates and in quadruplicates for 384-well plates.

### Image processing for on chip data analysis

For on chip assays, brightfield and red fluorescence images were initially processed by ImageJ v.1.50i. Cell counting was either performed manually or by Matlab v.R2015a based on the workflow shown on Supplementary Figure 2. Briefly, cells from brightfield and red fluorescence images were detected separately using a heuristic Hough Transformation model^28^ based on threshold implementation on circular diameter and pixel intensity. Next, each recognized cell was dissected into 10 × 10 pixels matrix for analysis of its pixel intensity. Subsequently, two layers of multi-radii analysis of pixel intensity around the center of each matrix distinguished signal from noise. Afterwards, cells were distinguished from noise based on the brightfield image, whereas cell viability was determined by signal intensity of corresponding red fluorescence image. Finally, positional information (defined by row: x, and column: y) and size (defined by radius: r) of discrete image matrix was used to classify each cell as “live” or “dead”. The total number of cells in each image corresponding to each droplet formation well on chip was summarized as a table in CSV format for calculation of cell viability. To ease application, we have developed a graphical user interface (GUI) for running on Matlab. Codes are available from the corresponding author upon request.

### Cell viability calculation

For on chip assays, the number of cells was counted in brightfield and red fluorescence images from each well respectively. Cell viability was calculated as follows:

1 $$Cell\,viability=\frac{(Total\,cells-Dead\,cells)}{Total\,cells}\,$$

where *Total cells* and *Dead cells* referred to the total number of cells counted from brightfield and red fluorescence images, respectively.

For normalized cell viability, mean cell viability of all sample wells were normalized to mean cell viability of all negative control wells.

2 $$Normalized\,cell\,viability=\frac{Sample}{DMSO\,control}$$

where *Sample* and *DMSO control* represented mean cell viability of sample and negative control wells, respectively.

For microtiter plate assays, average relative fluorescence signal measured by plate reader from 6 reads of each well was used as raw data point. Cell viability was calculated as follows:

3 $$Cell\,viability=\frac{Sample-Blank}{DMSO\,control-Blank}\,$$

where *Sample* represented raw data points of each sample well, whereas *DMSO control* and *Blank* represented average raw data points of all DMSO control and Blank wells, respectively.

Bar graphs and line plots were drawn by GraphPad Prism 5.1. Scatter plots were drawn by R v.3.3.2 using custom scripts. Figures were prepared by assembling images, graphs and plots using Adobe^®^ Illustrator^®^ CS6 v.16.0.0.

## Results

### Improvement of microfluidic chip design and validation

**Figure 1:**
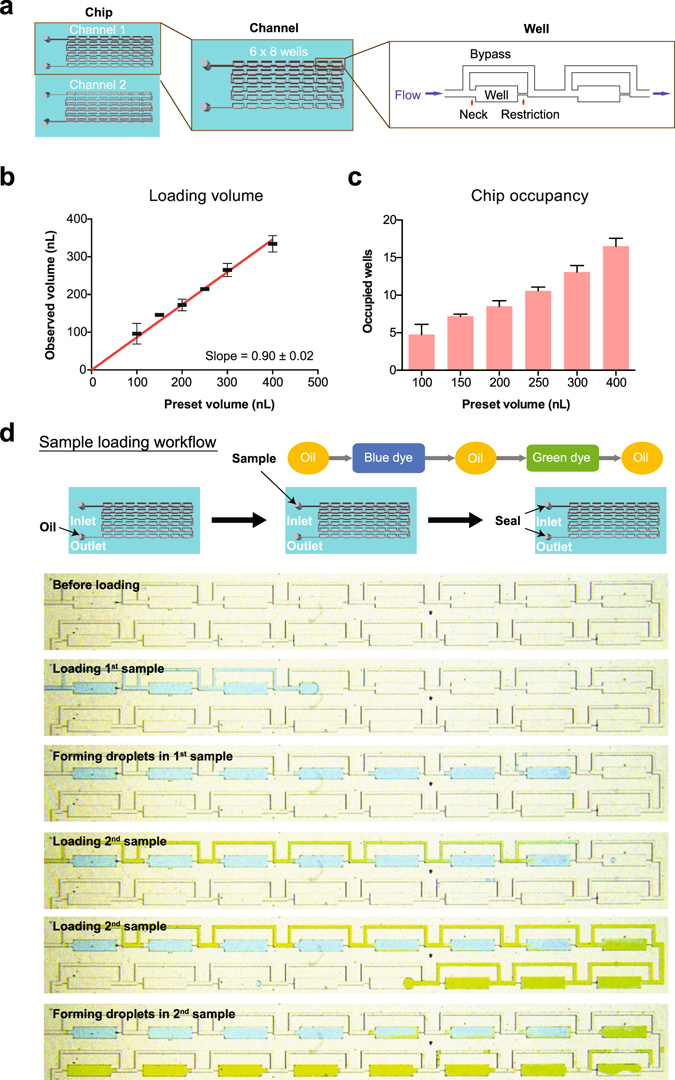
Microfluidic chip design and validation. (**a**) Each chip contained two channels comprising of an array of 6 × 8 wells. The channel facilitated fluid flow in one direction through the droplet formation well and bypass channel, while droplet was formed in the well due to restricted flow at the restriction; the neck prevented droplet escape and droplet coalescence with subsequent incoming aqueous fluid. (**b**) Correlation between observed volume (y-axis) and preset volume (x-axis) was plotted, where observed volume was calculated by multiplying the number of occupied wells (at 0.5 increments) by the theoretical well volume (length × width × height) of each well, whereas preset volume referred to the volume set on syringe pump; error bars denoted standard deviation of mean observed volumes obtained from all replicates for each preset volume. (**c**) Chip occupancy (y-axis) was plotted against preset volume (x-axis), where occupied wells was the number of occupied wells and preset volume referred to the volume set on syringe pump; error bars denoted standard deviation of the observed occupied wells in all replicates for each preset volume. (**d**) Two aqueous color dyes were loaded consecutively on chip to illustrate the sequential loading of different screening conditions in consecutive droplet formation wells. In this experiment, the food dyes were dissolved in water, representing the aqueous phase; the oil phase contained Fluorinert® FC-40 oil (Sigma-Aldrich, USA) supplemented with 2% 008-Fluorosurfactant (Ran Biotechnologies, USA). Firstly, we loaded the chip with oil. Next, food dyes of blue and green colors were segregated by translucent oil phase and loaded consecutively on chip. Each dye formed droplets in sequential order that was identical to the loading sequence.

In this study, we exploited a PDMS-based microfluidic chip design from previous studies^24^ for conducting cell-based drug screens. Our chip comprised of 2 channels of 6 rows of 8 chamber wells with adjacent bypass channels, forming an array of 48 droplet formation wells for each channel (Fig. 1a). Each chamber well consisted of a droplet formation well that was preceded by a neck and succeeded by a restriction, and a bypass channel (Fig. 1a). All fluids flowed in one direction and in sequential order as loaded in the inlet tubing (Supplementary Movie 1). Droplets were formed when aqueous solution filled the well and cut off from the bulk solution by subsequent incoming oil (Supplementary Movie 1).

However, distinct from previous design^24^, a neck was engineered before the droplet formation well to work as a droplet back flow restriction (Fig. 1a). The neck increased energy cost for droplet escape from the droplet formation well, especially during overnight incubation at elevated temperature of 37 °C as compared to room temperature of 25 °C during sample loading. In addition, the oil phase trapped in the neck helped to prevent the stored droplet from coalescence with subsequent incoming droplets in the main channel. The physical separation of droplets between wells was designed to prevent cross-contamination between drug treatment conditions in different droplets. Furthermore, the droplet formation wells also acted as reference grids for image acquisition. On the other hand, the width ratio of the neck to the bypass channel was optimized to 3:4 in order to favor fluid flow into the well over the bypass. In this work, three designs were tested: the widths of the neck to the bypass channel were 75 μm/100 μm, 100 μm/100 μm and 100 μm/150 μm, respectively. Results showed that the design of 75 μm/100 μm neck to bypass width favored fluid flow into the well over the bypass channel (Supplementary Movie 2). This ensured full well filling in order to maximize channel space usage.

Next, we tested whether our improved design facilitated robust droplet formation as reported. By loading with syringe pump, there was good correlation between the observed loading volume and the theoretical loading volume as preset on the syringe pump (Fig. 1b), implying that droplet formation was robust. Furthermore, the fidelity of observed volume as compared to preset volume suggested that the number of screen conditions could be adjusted. Given that each channel of our chip consisted of 48 wells, the total number of screen conditions could be calculated by dividing the total number of wells on chip by the number of wells loaded by a preset loading volume (Fig. 1c). For instance, we loaded 200 nL per sample in this study, which occupied 8–10 wells of our channel, so that 5 conditions were tested for one channel and 10 conditions for one chip. If the loading volume was reduced to 100 nL, the number of tested conditions would increase to 10 per channel and 20 per chip. The maximum number of screen conditions would be equal to the number of wells, i.e. 96 in this case. Because each treatment condition was loaded in discretion, the number of screen conditions could be flexibly adjusted based on sample input size and throughput requirement.

In order to minimize sample input and maximize sample usage, multiple drug treatment conditions must be tested on each channel. Hence, we used food dyes of different colors to testify the reliability of the chip in droplet formation and separation. In this experiment, the food dyes were dissolved in water, representing the aqueous phase; the oil phase comprised of Fluorinert® FC-40 oil supplemented with 2% 008-Fluorosurfactant. The sample loading workflow was illustrated in Fig. 1d. Firstly, we loaded the chip with oil. Next, food dyes of blue and green colors were segregated by translucent oil phase and loaded consecutively on chip. Each dye formed droplets in sequential order that was identical to the loading sequence (Fig. 1d and Supplementary Movie 1). No cross-contamination between droplets was observed unless coalescence occurred (Supplementary Figure 3a). Furthermore, the physical separation of droplets between wells prevented cross-contamination between samples in different droplets. Hence, these results demonstrated that testing multiple conditions in a single channel on chip is feasible.

Taken together, these results proved that our microfluidic chip is applicable for multi-drug conditions screening on a single channel with high flexibility based on sample input size and throughput requirement.

### Drug screening platform setup and optimization

After validation of our microfluidic chip, we tried to apply it to cell-based drug screening. The following criteria were considered: (1) the drug screening platform should maintain cell viability under investigated conditions; (2) the method is robust and well-controlled; (3) the readout is accurate and reproducible; and (4) the system is versatile for different cell culture systems.

Firstly, we optimized the oil phase to assure cell viability on chip for a minimum of 7 days for cancer cell lines. Commercial oils including Fluorinert^®^ series oils, silicone oil, and mineral oil in combination with different surfactants (either mixed with oil phase or aqueous phase) at various concentrations have been tested. Among these, Fluorinert^®^ FC-40 supplemented with 2% 008-Fluorosurfactant by Ran Biotechnologies provided optimal properties in terms of viscosity, volatility and droplet stability. Fluorosurfactant was added to prevent droplets from coalescence and cross-contamination of droplet contents when they touched each other (Supplementary Figure 3a). In addition, fluorosurfactant-emulsified droplets prevented fluorescent dye from adsorbing to the hydrophobic PDMS walls of the channel (Supplementary Figure 3b) or migrating into the oil phase (Supplementary Figure 3c), in order to maintain constant drug concentrations in droplets. Although droplet shrinkage due to evaporation after overnight incubation might affect drug concentration, we assumed that all droplets on the same chip had equal evaporation rate, so results should be comparable. This problem would be addressed in future design by continuous perfusion. On the other hand, the Droplet Generation Oil for Probes by Bio-Rad was also good for droplet generation, but its high volatility rendered it suboptimal for long-term cell culture on PDMS-based chips. Other oils and surfactant combinations were not used due to adverse properties: (1) the investigated oils in absence of surfactant provided poor droplet generation efficiency on our chip; (2) mineral oil had high viscosity that hindered its loading on chip; (3) surfactants like Triton X-100 and sodium dodecyl sulfate (SDS) compromised cell viability at all tested concentrations; and (4) the Pluronic^®^ series surfactants did not alter droplet generation efficacy, nor enhanced adherent cells to remain in suspension.

For the aqueous phase, applying optimal cell culture medium for cell survival is the priority. Next comes the consideration of droplet generation. In this study, three culture medium recipes, namely Dulbecco’s Modified Eagle Medium (DMEM), Advanced RPMI 1640 and StemMACS iPS-Brew XF medium, have been tested to be feasible for drug screening on chip. We hypothesized that other medium recipes should also be feasible because mammalian cells, in general, require similar ionic strength, which is a major consideration factor for chip performance. Fetal bovine serum (FBS) frequently used in mammalian cell culture possesses emulsification properties that affect droplet formation and stability. In this study, we found that 5% FBS was optimal, but empirical trial would always be recommended due to variance between products. Alternatively, additives should be added with caution. In our trial experiments, cell culture additives like 1% methyl cellulose, 0.1% Pluronic^®^ F-68, and 8 mg/mL Matrigel^®^ did not affect droplet formation (data not shown).

**Figure 2:**
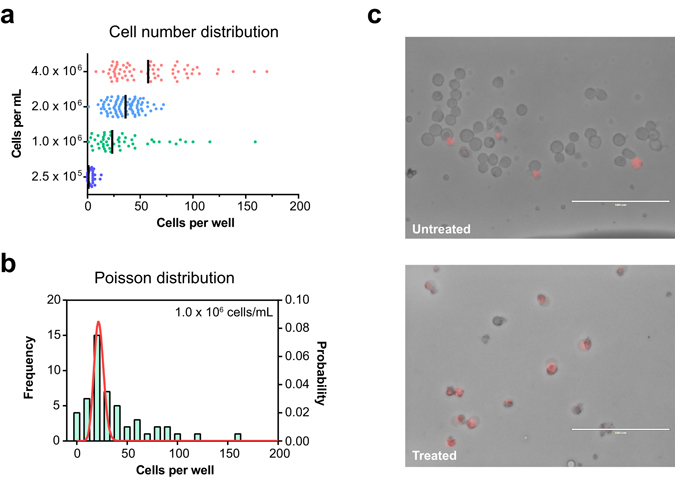
On chip drug screening platform setup and optimization. (**a**) Cell suspensions of different concentrations were loaded on chip (y-axis) and the total number of cells in each well (x-axis) was counted. MDA-MB-231 cells were used in all experiments, except for the 4.0 × 10^6^ cells/mL where Jurkat cells were used to avoid overcrowding. (**b**) Frequency plot of the total number of cells observed in each well prevailed good Poisson distribution at the cell concentration of 1.0 × 10^6^ cells/mL. (**c**) The cell-impermeable dye, ethidium homodimer 1, was used to stain dead cells after drug treatment. Dead cells were depicted red after merging brightfield image with red fluorescence channel (Ex. 531/40 nm, Em. 593/40 nm).

Secondly, we tested for optimal cell density on chip. Optimal cell density was considered based on two reasons: (1) sufficient cell population for statistical analysis of drug susceptibility, and (2) optimization of droplet cell density to avoid overcrowding. Overcrowded droplets led to cell aggregates, resulting in poor cell shredding during image processing and hence collapse of intelligent solution. By loading gradient concentrations of cells on chip, results manifested that there was positive correlation between the average number of cells per well and the concentration of cells before loading on chip (Fig. 2a). Nevertheless, as reported by other studies, Poisson distribution of cells per droplet prevailed in all wells regardless of the actual cell concentration used (Fig. 2b and Supplementary Figure 4). Consideration for cell concentration mainly involves cell size, which affects the volume occupied in each droplet. Consequently, 1 × 10^6^ cells/mL was used for MDA-MB-231 cells (average diameter ~20 μm) while 2 × 10^6^ cells/mL was used for Jurkat cells (average diameter ~ 10 μm). As for primary tumor dissociated cells, we applied cells at a concentration range of 1–3 × 10^6^ cells/mL and a loading volume of 200 nL for each treatment condition. This ensured a sample cell population of over 100 cells for each treatment condition without compromising cell counting due to cell aggregation. Taken together, our assay enabled flexible adjustment of cell concentration and loading volume to achieve predictable sample cell population for each screen condition. Generally, 1–2 × 10^6^ cells/mL works for the majority of mammalian cells.

Thirdly, we tested different cell viability indicator dyes for staining efficiency, indicator reliability and cytotoxicity on chip. One main drawback of our chip was: everything was premixed before loading on chip. Because of this reason, we tested for different cell viability indicator dyes and variant concentrations to ensure that the indicator dye had no impact on cell viability, at least within the time frame of the drug susceptibility test. Eventually, ethidium homodimer 1 staining was adopted to indicate dead cells. Ethidium homodimer 1 is a cell-impermeable, high affinity nucleic acid stain emitting strong red fluorescence after binding to DNA^29, 30^. It gave strong red fluorescence after cells were treated with drug but not DMSO (Fig. 2c). Time-lapse imaging of cells incubated with 2 μM ethidium homodimer 1 indicated that the dye did not affect cell death and proliferation within 48 h post-treatment (data not shown).

Lastly, we tried to make our drug screening platform versatile for different kinds of cancers. Because cells were suspended in droplets during drug treatment, adherent cells formed aggregates in the absence of physical support (Supplementary Figure 3d). Therefore, we tried to emulsify cells with detergents, maintain cells in semi-solid matrix using Matrigel^®^, increase fluid viscosity using sucrose, *etc*, but failed. Eventually, we found that culture medium supplemented with 1% methyl cellulose maintained cell viability on chip and did not affect cell proliferation off chip for both suspended and adherent cells (Supplementary Figure 3e). Methyl cellulose has been applied to grow 3D organoid cultures of human pluripotent stem cells (HPSCs) *in vitro* ^31^. Our observation of its success was reduced cell movement inside droplets, thus preventing cells from touching and maintained them in suspension. Hence, we used this condition for subsequent drug screening experiments.

### Drug screening of suspended and adherent cancer cell lines

**Figure 3:**
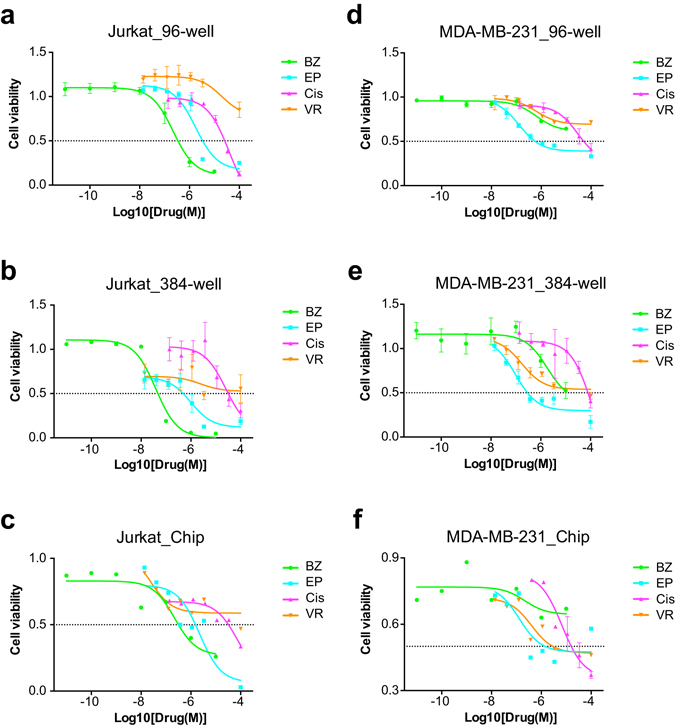
Four drugs, namely Bortezomib (BZ), Epirubicin (EP), Cisplatin (Cis), and Vorinostat (VR) were used to screen Jurkat cells (**a**–**c**) and MDA-MB-231 cells (**d**–**f**), as models for suspended and adherent cancer cells respectively, on three drug screening platforms using 96-well plate, 384-well plate and our chip assay. A horizontal dashed line was drawn at 50% cell viability for comparison of IC_50_ between different screening methods. All graphs were plotted by cell viability (y-axis) against the log of final drug concentration (x-axis); error bars denoted standard deviation of mean cell viability obtained from all replicates in parallel experiments, except for the chip assay of Cisplatin-treated MDA-MB-231 cells (**f**) where error bars denoted standard deviation of mean cell viability obtained from all droplets of two independent experiments.

Next, we performed drug screening experiment on Jurkat cells and MDA-MB-231 cells, as models for suspended and adherent cells, respectively. We tested these two cell lines against four anti-cancer drugs, namely Bortezomib, Epirubicin, Cisplatin and Vorinostat (Supplementary Table 1). Comparison of the chip screen results of both cell lines with those obtained from conventional plate reader assays using 96-well and 384-well microtiter plates manifested qualitative assessment of drug efficacy regardless of drug screening platform (Fig. 3), suggesting that our chip assay could be used to qualitatively assess whether a drug is effective or ineffective for the tumor tested *in vitro*. On the other hand, quantitative assessment of drug potency across the drug panel should be done on a single drug screening platform, in order to eliminate system errors and assay differences between platforms. Nevertheless, given that *in vitro* assays do not consider about pharmacokinetic differences between drugs *in vivo*, these results should be combined with clinical expertise to finalize therapeutic decision.

In this experiment, ranking of drugs under all three assays using 96-well plate (Fig. 3a), 384-well plate (Fig. 3b) and our microfluidic chip (Fig. 3c) were relatively consistent for Jurkat cells. In consideration of IC_50_ values and lowest cell viability under highest drug dose tested, the ranking demonstrated that Jurkat cells were most susceptible to Bortezomib, followed by Epirubicin and then Cisplatin, whereas Vorinostat treatment showed a plateau above 50% cell viability under all doses tested in all three assays (Fig. 3a–c). However, if we directly compared dose response curves across the screening platforms, there was less consistency between the curves (Supplementary Figure 5a–d). For instance, in Jurkat cells treated with Bortezomib, there was a prominent left shift of the dose response curve of 384-well plate screen (IC_50_ = 39 nM) as compared to the 96-well plate screen data (IC_50_ = 22.1 μM), whereas on chip screen data (IC_50_ = 22.1 μM) prevailed higher conformity to 96-well plate screen (Supplementary Figure 5a). Dose response curves of Epirubicin treatment of Jurkat cells were more similar, with an IC_50_ span of 1.0–2.2 μM on all three platforms (Supplementary Figure 5b). Cisplatin treatment of Jurkat cells yielded almost identical response in Jurkat cells in both 96-well and 384-well plate assays (both IC_50_ = 39.0 μM), whereas our chip assay gave identical IC_50_ value of 39.0 μM but the dose response curve had smaller amplitude (Supplementary Figure 5c). Lastly, Jurkat cells were least susceptible to Vorinostat treatment (all IC_50_ > 50.0 μM), and this response was consistent in both plate reader assays and on chip (Supplementary Figure 5d). Taken together, our chip assay provided identical drug ranking assessment for Jurkat cells across the drug panel used in this study as compared to conventional plate reader assays.

Alternatively, MDA-MB-231 cells demonstrated less consistency in drug ranking among assays using 96-well plate (Fig. 3d), 384-well plate (Fig. 3e) and our microfluidic chip (Fig. 3f). Epirubicin prevailed consistently lower IC_50_ values towards MDA-MB-231 cells in all three assays, depicting IC_50_ values of 0.6 μM and 0.5 μM for 96-well plate (Fig. 3d) and 384-well plate (Fig. 3e) assays respectively, whereas cell viability leveled off at approximately 50% on chip after reaching its IC_50_ of 5.5 μM (Fig. 3f). The other three drugs exhibited more diverse effects on MDA-MB-231 cells among different platforms (Fig. 3d–f). This trend was also observed in the lower curve conformity among the three assays under treatment of MDA-MB-231 cells with Bortezomib (Supplementary Figure 5e), Cisplatin (Supplementary Figure 5g) and Vorinostat (Supplementary Figure 5h), as compared to Epirubicin (Supplementary Figure 5f). For instance, MDA-MB-231 cells responded mildly to Bortezomib, with IC_50_ values of over 10.0 μM on chip and on 96-well plate, whereas 50% cell viability was observed at 10.0 μM Bortezomib dose on 384-well plate (Supplementary Figure 5e). Cisplatin showed IC_50_ values of over 50.0 μM in both 96-well and 384-well plates for MDA-MB-231 cells, whereas the cells showed 10-fold higher sensitivity towards this drug (IC_50_ = 4.2 μM) on chip (Supplementary Figure 5g). Vorinostat showed a plateau above 50% cell viability under 96-well plate and 384-well plate assays, while its IC_50_ was 33.3 μM on chip (Supplementary Figure 5h). Taken together, there was discrepancy on drug ranking among all of the three drug screening platforms for MDA-MB-231 cells. Because MDA-MB-231 cells were cultured in adhesion on both 96-well and 384-well plates, and in suspension on chip, where the same culture medium recipe was used for all three assays carried out in parallel, we deduced that variant drug response was not attributed by culture condition and/or screening platform. Instead, we hypothesized that inherent cell heterogeneity of this cell line attributed to difference in drug response. Nevertheless, the most effective drug Epirubicin was conserved in all three assays, suggesting that our chip was capable of indicating effective drug(s) from the drug panel even in heterogeneous cell population.

**Figure 4:**
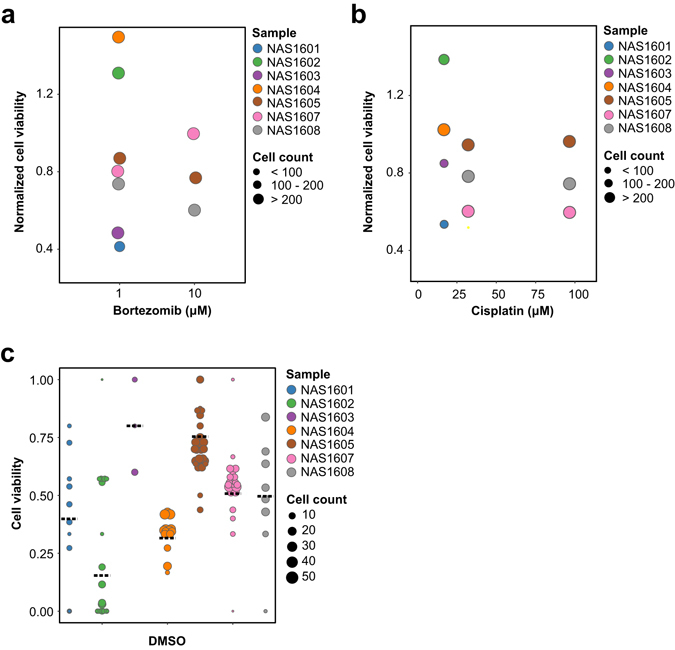
Seven primary nasopharyngeal tumors were screened against two drugs and mock treatment control with DMSO. (**a**) Primary tumor dissociated cells were screened against 1 μM and/or 10 μM Bortezomib (x-axis) for 16–24 h. Normalized cell viability (y-axis) referred to cell viability normalized to DMSO-treated control; each dot indicated the mean cell viability of all droplets obtained from one tumor sample, whereas each tumor sample was represented by one color, and the size of the dot denoted the total sample cell population in all droplets of the specified tumor sample. (**b**) Primary tumor dissociated cells were screened against 20 μM, 33.3 μM, and/or 100 μM Cisplatin (x-axis) for 16–24 h. Normalized cell viability (y-axis) referred to cell viability normalized to DMSO-treated control; each dot indicated the mean cell viability of all droplets obtained from one tumor sample, whereas each tumor sample was represented by one color, and the size of the dot denoted the total sample cell population in all droplets of the specified tumor sample. (**c**) Observed cell viability (y-axis) of all primary tumor samples (x-axis) reported in this study after 16–24 h mock treatment was depicted; each dotted line indicated the mean cell viability of all droplets obtained from one tumor sample, whereas each dot indicated the mean cell viability of all cells in one droplet, each tumor sample was represented by one color, and the size of the dot denoted the sample cell population size in one droplet.

**Figure 5:**
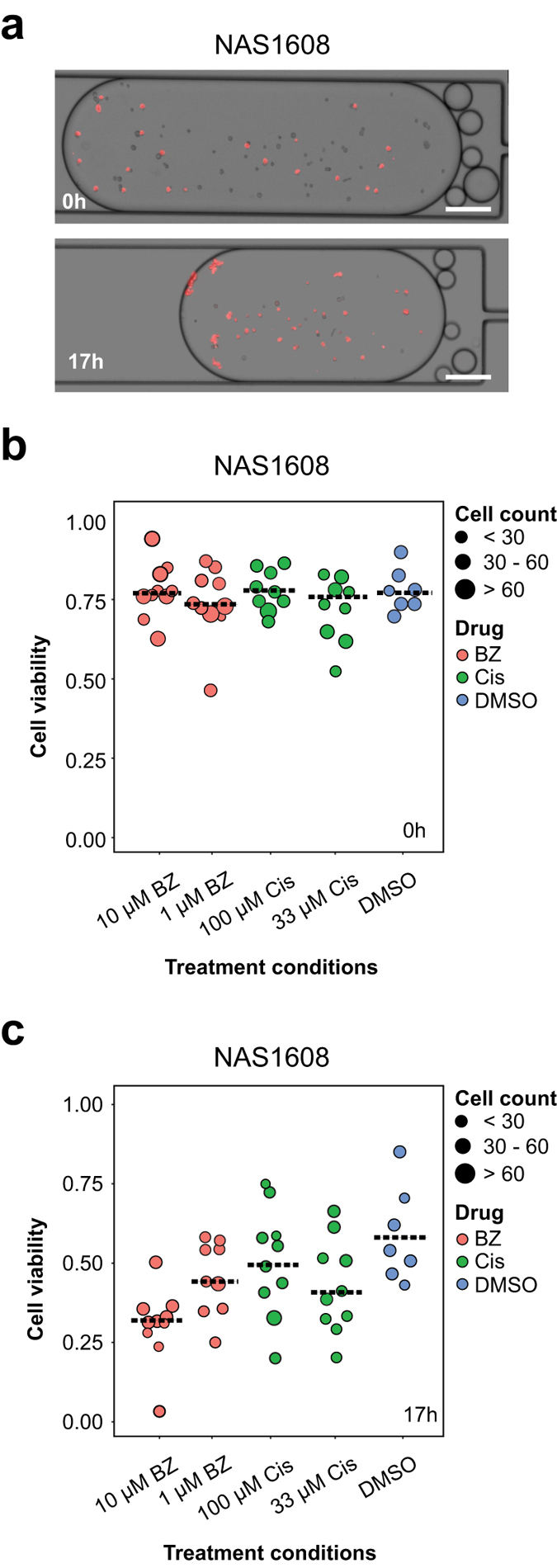
One human nasopharyngeal cancer sample NAS1608 was shown as an example. (**a**) Merged images between brightfield and red fluorescence channel (Ex. 531/40 nm, Em. 593/40 nm) were taken before and after 10 μM Bortezomib treatment for 17 h. Droplets were maintained but their volume shrank due to water evaporation inside the humidified CO_2_ incubator. Scale bar indicates 25 μm. (**b**) Observed cell viability (y-axis) was plotted against different treatment conditions (x-axis) in human nasopharyngeal cancer sample NAS1608 before treatment. (**c**) Observed cell viability (y-axis) was plotted against different treatment conditions (x-axis) in human nasopharyngeal cancer sample NAS1608 after 17 h treatment. For both cell viability graphs, each dotted line indicated the mean cell viability of all droplets under the specified treatment conditions, whereas each dot indicated the mean cell viability of all cells in one droplet, and the size of the dot denoted the sample cell population size in one droplet. BZ denoted Bortezomib; Cis denoted Cisplatin.

Close inspection of the dose response curves between different screening platforms prevailed overall smaller amplitude in the chip screen curves as compared to the plate reader assay curves (Supplementary Figure 5). This might be conferred by exploitation of single cell counting for cell viability assessment on chip, as compared to bulk population measurement of cellular metabolic activity on plate. In this aspect, our chip provided a preliminary tool to observe differential drug response in single cells, without compromising population analysis using multiple droplets for each drug treatment condition. Results showed that although cell viability differed vastly between individual droplets (Figs 4 and 5), analysis of the whole sample cell population in all droplets compensated for this variance and yielded reproducible results. For example, screening of MDA-MB-231 cells against Cisplatin demonstrated comparable mean cell viability between two independent experiments, the standard deviation of which might be smaller than that of replicates in parallel experiments in 96-well or 384-well plates (Supplementary Figure 5g). Nevertheless, our current readout method is still crude, so we are yet to understand the underlying mechanism of differential drug response in single cells.

In conclusion, our chip assay provided qualitative drug potency assessment in MDA-MB-231 cells. It provided drug ranking assessment of Jurkat cells against the four anti-cancer drugs used in this study, which was comparable to that obtained from 96-well and 384-well plate reader assays.

### Drug screening of primary tumor dissociated cells from human patients

Lastly, we screened seven primary nasopharyngeal tumors from human patients using our microfluidic chip assay. The tumors were collected by surgical resection from human cancer patients. The seven tumors varied in size from 0.2 cm to 0.5 cm in diameter and yielded variable cell numbers for use in this study from 0.5–1.0 × 10^5^ cells using identical tumor dissociation technique. Given the limited number of cells obtained from human primary tumors, parallel drug screening on 96-well or 384-well plates could be not be attained due to their requirement for higher cell numbers (Table 2). Consequently, all tumor samples were screened on chip against two anti-cancer drugs, namely Bortezomib and Cisplatin, and DMSO control for up to 5 conditions. Cells were treated for a total of 16–24 h and cell viability was measured by ethidium homodimer 1 staining.

**Table 2:** Comparison between different drug screening platforms.

	96-well plate	384-well plate	PDMS chip
Format	8 × 12 wells	16 × 24 wells	2 channels of 6 × 8 wells each
Well bottom	Round, flat	Square, flat	N/A
Cell growth area	0.32 cm^2^	0.06 cm^2^	N/A
Culture medium per well	50 μL	30 μL	21.39–26.91 nL
Cells per well	5.0 × 10^5^ cells	1.0 × 10^5^ cells	10–100 cells
Cell density	15.6 × 10^5^ cells/cm^2^	16.6 × 10^5^ cells/cm^2^	N/A
Cell viability assay	Alamar Blue	Alamar Blue	Ethidium homodimer 1 staining
Viability assessment	Bulk population	Bulk population	Single cells
Measurement	Plate reader	Plate reader	Fluorescence microscope
Measurement time	3 minutes	5 minutes	1 h (manual) 30 min (automatic)
Cell loading time	1 h (manual) 10–20 min (robot)	1.5 h (manual) 20–30 min (robot)	5 min (manual)? (automatic)
Throughput	8 drugs, 4-dose, 3 replicates	24 drugs, 4-dose, 4 replicates	5 drugs, 1-dose, single cell replicates
Cells per drug per dose	1.5 × 10^6^ cells	4.0 × 10^5^ cells	16,000 cells (current) 100 cells (potential)
Cost per dose (without drug)	HKD1.0	HKD0.6	HKD0.2

Mock treatment of the seven human primary tumor dissociated cells showed diverse cellular response after 16–24 h treatment with DMSO alone (Fig. 4c). The range of cell viability among individual droplets within one sample also differed vastly, from 100% in the samples of NAS1602 and NAS1607 to the smallest range of 26.2% observed in NAS1604 sample (Fig. 4c). Of note, NAS1603 and NAS1605 exhibited over 70% mean cell viability, whereas the majority of primary tumor samples showed approximately 50% mean cell viability, and NAS1602 only retained 16.4% of viable cells after overnight incubation (Fig. 4c). Explanation of the diverse observed cell viability remained elusive.

Next, we compared the chip screen data of all seven human primary nasopharyngeal tumor samples against two anti-cancer drugs, namely Bortezomib and Cisplatin, respectively (Fig. 4a and b). In order to illustrate difference in drug susceptibility among the seven human nasopharyngeal tumor samples, we normalized the drug treatment data of all samples to that of mock treatment to obtain normalized cell viability (Fig. 4a and b). Results showed diverse response of the primary tumors towards the two drugs, with no explicit correlation to cellular response towards DMSO treatment in parallel experiments on the same chip (Fig. 4a–c). Nevertheless, dose-dependent reduction of cell viability was observed in Bortezomib treatment of two nasopharyngeal tumor samples, namely NAS1605 and NAS1608 (Fig. 4a). However, inverse correlation was observed in NAS1607 under Bortezomib treatment (Fig. 4a), while its susceptibility leveled off under 100 μM and 33 μM Cisplatin treatment (Fig. 4b). On the other hand, correlation between Cisplatin concentration and cell viability was less prominent in all seven human primary tumor samples (Fig. 4b). Furthermore, we observed more fluctuations in normalized cell viability when it was close to 1.0, hypothesized to incur from subtle difference between single cells in the population. There was no conclusive explanation to any of the observed viability variation, given that the only amplified tumor NAS1604 exhibited 34.1% mean cell viability after overnight DMSO treatment (Fig. 4c). The low tumor amplification rate suggested of further optimization of our culture conditions for tumor amplification.

Drug susceptibility between the primary tumor NAS1604 and its derived cell line NAS1604C was compared (Supplementary Figure 6). NAS1604 primary tumor dissociated cells exhibited highest mean cell viability under 1 μM Bortezomib treatment as compared to DMSO treatment, whereas 20 μM Cisplatin treatment exhibited lowest mean cell viability (Supplementary Figure 6a). Coincidently, the tumor cells amplified by 2D monolayer culture, named as NAS1604C, also exhibited highest cell viability under 1 μM Bortezomib treatment as compared to mock treatment with DMSO, whereas dose-dependent response was observed in NAS1604C towards Cisplatin (Supplementary Figure 6b). Revision of cellular drug response in individual droplets of NAS1604 demonstrated a larger range of droplet cell viability of 64.0% under Cisplatin treatment, as compared to droplets treated with Bortezomib (42.6%) or DMSO (26.2%) (Supplementary Figure 6a). Here, the concept of droplet cell viability was introduced to indicate the total number of live cells among all cells in each individual droplet; in turn, the range of droplet cell viability referred to the range of cell viability of all droplets under specified treatment condition. For instance, two droplets depicted 69.7% (n = 44/66) and 5.7% (n = 2/35) of maximum and minimum droplet cell viability, respectively, among all droplets under 20 μM Cisplatin treatment in NAS1604 cells, resulting in 64.0% droplet cell viability range. In contrast to the primary tumor NAS1604, the droplet cell viability range among droplets treated with 1 μM Bortezomib (37.5%) and 11 μM Cisplatin (36.5%) in the derived cell line NAS1604C was smaller than corresponding conditions in the primary tumor sample NAS1604, whereas treatment of NAS1604C cells with 33 μM Cisplatin (71.6%) or DMSO (83.3%) depicted larger droplet cell viability (Supplementary Figure 6b). However, close inspection of each treatment condition in the derived cell line NAS1604C showed one droplet outlier under the treatment conditions of 1 μM Bortezomib, 33 μM Cisplatin and DMSO (Supplementary Figure 6b), removal of which yielded droplet cell viability range of 16.1%, 40.8% and 25.0%, respectively, that were smaller than corresponding conditions in the primary tumor NAS1604. This observation suggested that the NAS1604 cells became more homogeneous during cell line derivatization. Indeed, the derived cells were fibroblast-like (Supplementary Figure 6d), whereas the Hematoxylin and Eosin (H&E) stained tumor tissue sections showed that the primary tumor NAS1604 was undifferentiated nasopharyngeal tumor (Supplementary Figure 6c), indicating that cell morphology changed during cell derivatization. Nonetheless, the molecular mechanism remained obscure. On the other hand, mean cell viability of all treatment conditions were higher in the derived cell line NAS1604C than corresponding conditions in the primary tumor NAS1604, reminiscent of increased cell viability that was required for tumor amplification.

Collectively, these data reflected that: (1) primary tumors had diverse susceptibility towards different drugs, thus supporting for the need for personalized cancer therapy; and (2) primary tumors before and after *in vitro* amplification might prevail similar drug susceptibility, while their morphological cell type might be different.

In order to exemplify the capability of our microfluidic chip, one nasopharyngeal tumor sample was shown in details as an example (Fig. 5). First, cell imaging immediately after chip loading showed a certain extent of ethidium homodimer 1-labelled cells (Fig. 5a), which was roughly consistent to Trypan Blue staining results of 37% measured by cell counter (data not shown). Cell viability measurement by ethidium homodimer 1 staining at 0 h showed no significant difference in mean cell viability among different treatment conditions (Fig. 5b). However, mean cell viability of NAS1608 cells declined from 75.8% to 56.3% before and after 17 h DMSO treatment (Fig. 5b and c). Decrease in mean cell viability from 78.8% to 34.6% and from 72.9% to 42.5% was observed after 17 h of 10 μM and 1 μM Bortezomib treatment respectively (Fig. 5b and c). Cisplatin treatment led to a reduction of mean cell viability of 23.4% and 27.7% under the concentrations of 100 μM and 33 μM, respectively (Fig. 5b and c). Normalization to DMSO-treated negative control (n = 264) and calibration by 0 h treatment data showed that 17 h treatment of NAS1608 cells with 10 μM Bortezomib resulted in 51.7% mean cell death (n = 582), while 100 μM Cisplatin led to 21.0% mean cell death (n = 427). Statistical analysis of the drug response of NAS1608 cells towards Bortezomib and Cisplatin showed significant difference in two-way ANOVA, whereas Tukey’s honest significant difference (HSD) test confirmed the difference between 10μM Bortezomib and DMSO treatment for NAS1608 human primary nasopharyngeal tumor sample (Supplementary Table 2).

Further investigation into drug response among individual droplets showed stark contrast before and after overnight incubation (Fig. 5b and c). Before treatment, the range of droplet cell viability was 29.0%, 31.1% and 16.2% under treatment with 10 μM Bortezomib, 100 μM Cisplatin and DMSO, respectively (Fig. 5b). 17 h post-treatment demonstrated a droplet cell viability range of 49.7%, 56.5% and 50.5% under treatment with 10 μM Bortezomib, 100 μM Cisplatin and DMSO, respectively (Fig. 5c), Thus, difference in the range of droplet cell viability before and after DMSO treatment was largest (34.2%), suggesting that some cells might have responded to mock treatment while others did not. To dissect this phenomenon, we looked at individual droplets before and after treatment. In 5 out of 8 wells (n = 207), DMSO induced less than 20% decrease in droplet cell viability, whereas 1 well (n = 53) showed 27.0% decrease in droplet cell viability, and 2 wells (n = 57) showed over 30% decrease in droplet cell viability. Hence, droplets that exhibited droplet cell viability reduction of over 30% under DMSO treatment contained merely 21.6% of the total sample cell population (n = 57/264). In contrast, 10 μM Bortezomib treatment reduced droplet cell viability by a minimum of 29.3% (n = 44), and a maximum of 62.9% (n = 74), whereas the majority of droplets exhibited a droplet cell viability reduction of 35–55% (n = 464). Hence, droplets that prevailed droplet cell viability reduction of over 30% under 10 μM Bortezomib contained 92.4% of the total sample cell population (n = 538/582). Taken together, our data suggested that NAS1608 cells responded to 10 μM Bortezomib with higher cell numbers and conformity as compared to DMSO, thus giving significant difference to Bortezomib treatment as compared to DMSO treatment.

In conclusion, our data demonstrated that mean cell viability could be used to reveal the percentage of cells that responded to the investigated drug(s), applied to qualitative drug potency assessment and drug ranking. On the other hand, the range of droplet cell viability suggested the conformity of cellular drug response towards the investigated drug(s).

## Discussion

In this study, we used a centimeter-sized PDMS-based droplet microfluidic chip to provide efficient evaluation of drug susceptibility of cancers. Our data indicated that our system could be used to screen as few as 16,000 cells obtained from primary cancer for each treatment condition within 24 h after tumor resection from cancer patients. Rapid screening for effective therapy is virtuous, especially for fast-growing cancers from the pancreas (20.8%), lung (32.1%), brain (40.1%) and oesophagus (41.9%), which kills patients within one year after diagnosis^32^. Our current assay provided empirical evidence for rapid drug potency assessment within 24 h. This would allow clinical doctors to determine their patient’s therapeutic regime within 2 days. Furthermore, the cost of our chip was merely HKD0.20 per chip (Table 2), making it pragmatically affordable for all cancer patients. Hence, our technology provided unprecedented opportunity for rapid evidence-based decision making for personalized cancer therapy.

Our microfluidic chip was improved from the design of our previously reported PDMS-based microfluidic chip^24^, the design of which met our requirement for system robustness and assay compatibility. Utilization of a similar design on drug susceptibility test of MCF-7 cells was reported recently^33^. However, their pipette loading method restricted application on high throughput screening. More importantly, cell lines were prone to survive and proliferate on chip, whereas cells dissociated from primary tumor died quickly under mock treatment after 16–24 h treatment (Fig. 4c). Therefore, we limited drug treatment time to 24 h for human primary tumor dissociated cells.

Except for cancers of the blood where cancer cells could be obtained by liquid biopsy, solid tumor biopsies have been difficult to obtain. Thus, we used surgically resected tumors for this study. Nevertheless, Jonas, *et al*., reported *in situ* screening of superficial mammary tumors by an implanted microfluidic device^20^. However, this device has only been tested on T2 breast cancer, which is defined as tumors of 2 to 5 cm in diameter in absence of metastasis to auxiliary lymph nodes. In comparison, we obtained nasopharyngeal tumors of 0.2 to 0.5 cm in diameter, which was 10-fold smaller in size. Xu, *et al*., reported screening of primary tumor dissociated cells from Stage IA lung cancer patients^18^, the tumor size of which was closer to this study. In their study, three gradient concentrations mixed by diffusion were screened on each channel, and four channels were fabricated on one chip^18^. Because the construction of their chip was fixed, neither the sample input size nor drug dose could be adjusted. Similarly, our chip had limitation on the number of wells fabricated on each channel, but our assay provided flexibility on sample input size and screen throughput by adjusting either loading volume or cell concentration, or both. In addition, drug concentration could be freely adjusted during premixing with cells before loading on chip. Nevertheless, we have not yet developed our assay to reach its maximum screen throughput. The major obstacle was limitation of instrumentation, including the lack of an autosampler for sample loading and a fluorescence microscope equipped with an automated stage for image acquisition. Hence, our chip assay demonstrated in this study could only provide a flexible tool for rapid, low-input drug screening of primary cancers at a reasonable throughput.

Ethidium homodimer 1 staining was used to measure single cell viability in this assay. Our data supported for observation of differential drug response in cancer cell lines and primary tumor dissociated cells from human cancer patients. On the population scale, mean cell viability obtained on chip could be applied to assessing drug potency and ranking drugs in a drug panel. Additionally, the conformity of drug response could be implied from the range of droplet cell viability. Nevertheless, our readout method provided no clue to understanding the mechanism of drug susceptibility differences between samples on chip. Fluidigm Polaris^TM^ provides a platform for single cell drug treatment followed by RNA sequencing library preparation for single cells. However, with only 48 cells on chip, Polaris^TM^ lacks the statistical power of population analysis that is essential for heterogeneous tissue samples. Thus, investigation on the combination of our chip with other techniques, for example, next generation sequencing (NGS), fluorescence activated cell sorting (FACS), *etc*, needs to be explored in order to elucidate the molecular background of the investigated cancer.

In conclusion, our microfluidic chip assay provides a powerful tool for rapid, low-input drug screening of primary cancers. Adaptation of the assay to suspended and adherent cancer cell lines suggests of its application in potentially all types of cancers. It provides us the opportunity to observe and quantify cellular drug response on the single cell level, whereas population analysis is achievable by statistical analysis of multiple droplets.

## Electronic supplementary material

Supplementary Movie 1

Supplementary Movie 2

Supplementary Information
