# Author Correction: Drug screening of cancer cell lines and human primary tumors using droplet microfluidics

**DOI:** 10.1038/s41598-019-55120-y

**Published:** 2019-12-04

**Authors:** Ada Hang-Heng Wong, Haoran Li, Yanwei Jia, Pui-In Mak, Rui Paulo da Silva Martins, Yan Liu, Chi Man Vong, Hang Cheong Wong, Pak Kin Wong, Haitao Wang, Heng Sun, Chu-Xia Deng

**Affiliations:** 1 Cancer Centre, Faculty of Health Sciences, University of Macau, Macau, China; 2 State-Key Laboratory of Analog and Mixed-Signal VLSI (AMSV), University of Macau, Macau, China; 3 Department of Electrical and Computer Engineering, Faculty of Science and Technology, University of Macau, Macau, China; 4 Instituto Superior Técnico, Universidade de Lisboa, Lisboa, Portugal; 5 Department of Computer and Information Science, Faculty of Science and Technology, University of Macau, Macau, China; 6 Department of Electromechanical Engineering, Faculty of Science and Technology, University of Macau, Macau, China

Correction to: *Scientific Reports* 10.1038/s41598-017-08831-z, published online 22 August 2017

This Article contains errors in the Results section.

“On the other hand, the width ratio of the neck to the bypass channel was optimized to 3:4 in order to favor fluid flow into the well over the bypass. In this work, three designs were tested: the widths of the neck to the bypass channel were 75 μm/100 μm, 100 μm/100 μm and 100 μm/150 μm, respectively. Results showed that the design of 75 μm/100 μm neck to bypass width favored fluid flow into the well over the bypass channel (Supplementary Movie 2).”

should read:

“On the other hand, the width ratio of the bypass to the neck channel was optimized to 3:4 in order to favor fluid flow into the well over the bypass. In this work, three designs were tested: the widths of the bypass to the neck channel were 75 μm/100 μm, 100 μm/100 μm and 100 μm/150 μm, respectively. Results showed that the design of 75 μm/100 μm bypass to neck width favored fluid flow into the well over the bypass channel (Supplementary Movie 2).”

