# Supplementary material for: Drug screening of cancer cell lines and human primary tumors using droplet microfluidics
Source: Sci Rep. 2017 Aug 22;7:9109. doi: 10.1038/s41598-017-08831-z (PMC5567315; doi:10.1038/s41598-017-08831-z)
Supplement: Supplementary file 3 — Supplementary Information [file 41598_2017_8831_MOESM3_ESM.pdf]

Drug screening of cancer cell lines and human primary tumors using droplet microfluidics

Ada Hang-Heng Wong[1], Haoran Li[2], Yanwei Jia[2], Pui-In Mak[2,3], Rui Paulo da Silva Martins[2,3,4], Yan Liu[5], Chi Man Vong[5], Hang Cheong Wong[6], Pak Kin Wong[6], Haitao Wang[1], Heng Sun[1], Chu-Xia Deng[1,#].

Affiliation(s):

[1] Cancer Centre, Faculty of Health Sciences, University of Macau, Macau, China.

[2] State-Key Laboratory of Analog and Mixed-Signal VLSI (AMSV), University of Macau, Macau, China.

[3] Department of Electrical and Computer Engineering, Faculty of Science and Technology, University of Macau, Macau, China.

[4] On leave from Instituto Superior Técnico, Universidade de Lisboa, Portugal.

[5] Department of Computer and Information Science, Faculty of Science and Technology, University of Macau, Macau, China.

[6] Department of Electromechanical Engineering, Faculty of Science and Technology, University of Macau, Macau, China.

[#] This indicates correspondence author(s).

Correspondence should be addressed to:

Name: Chu-Xia Deng, PhD

Phone: +853 - 8822 4997

Email: cxdeng@umac.mo

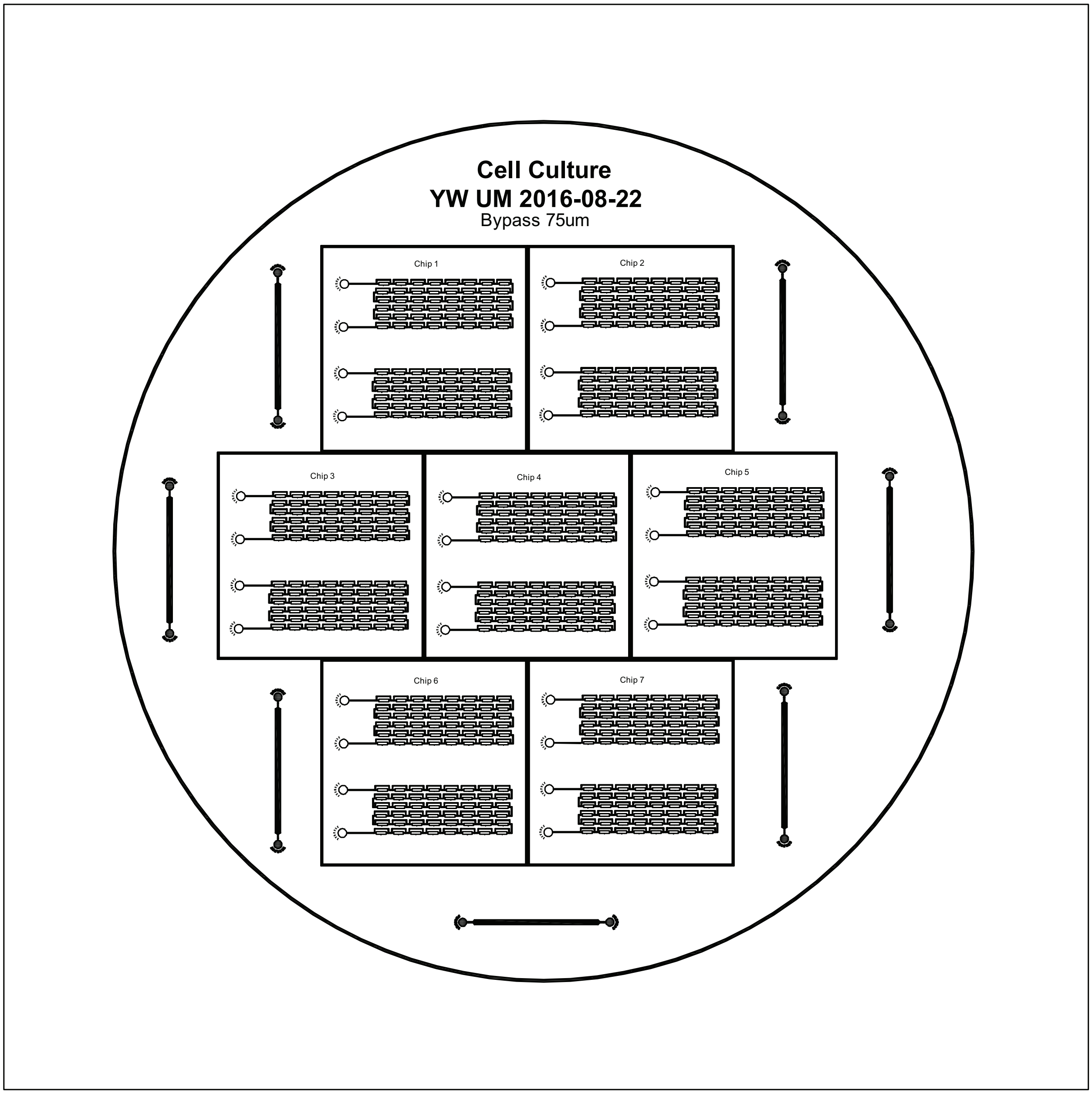

Supplementary Figure 1. The photomask for silicon wafer fabrication by soft lithography was shown. The square photomask was 127 × 127 mm in size, while the circular silicon wafer had a diameter of 100 mm. Each wafer contained 7 drug screening chips and 7 single channels. Each drug screening chip comprised of 2 channels of 6 × 8 wells, with one inlet and one outlet, respectively, at each end of the channel. Each single channel contained one inlet and one outlet at each end, respectively, for testing conditions. The drawing was completed using AUTODESK® AutoCAD® software.

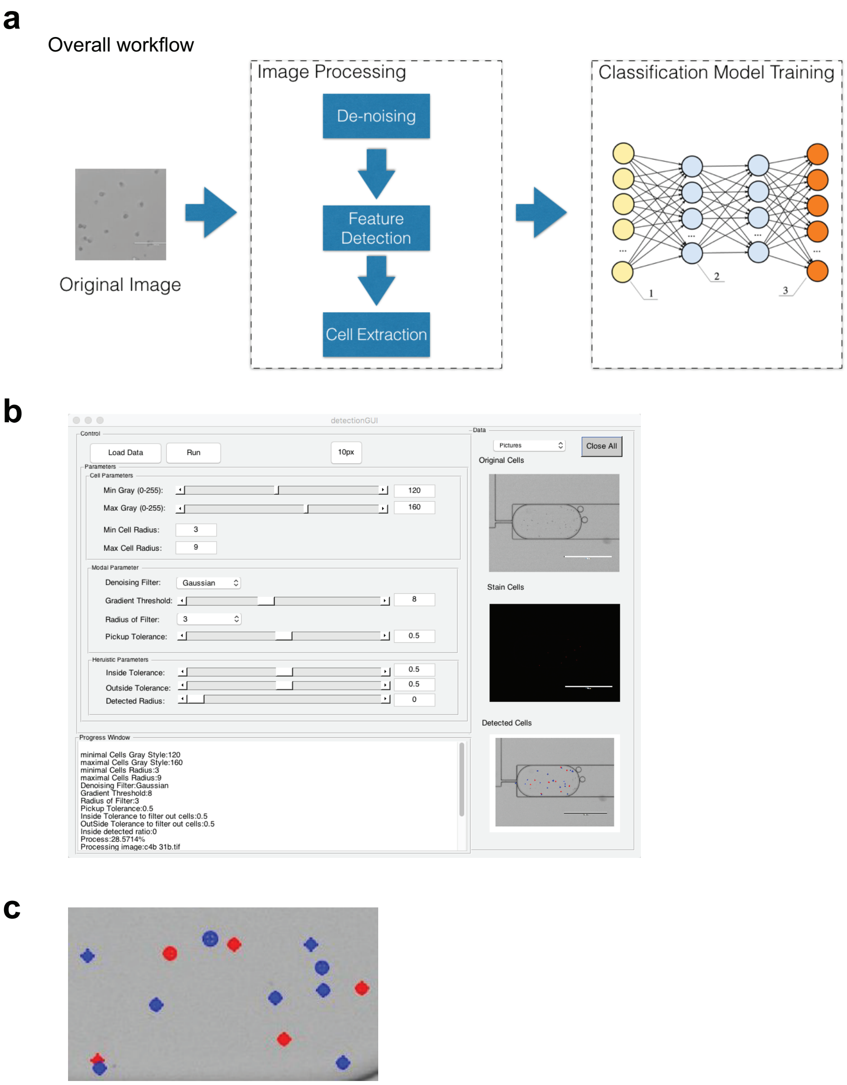

Supplementary Figure 2. Automatic cell counting was applied to analyze on chip drug screening data. (a) Overall workflow of the cell counting algorithm was summarized. Briefly, the original brightfield image was first de-noised, then cells were extracted by features like circular diameter and pixel intensity, and finally classified into brightfield only (live cells) and dual channel (dead cells) based on each cell's positional information (defined by row: x, and column: y) and size (defined by radius: r). (b) The graphical user interface (GUI) of our Matlab algorithm was shown. (c) Cells extracted by the algorithm were encircled in blue (live cells) or red (dead cells) and shown in the "Detected Cells" window of our GUI.

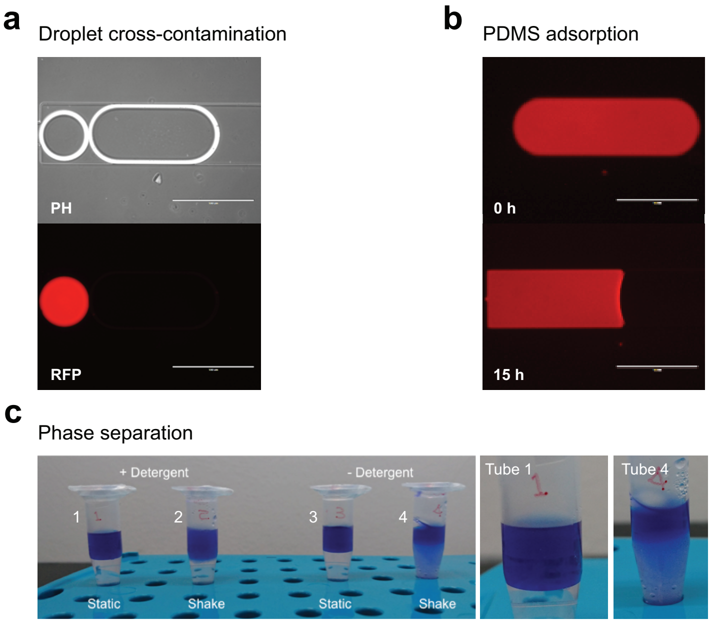

Chemical compound	Formula weight (Da)	logP	Reference
Cisplatin	298.035	-2.19	DrugBank
Bortezomib	384.237	0.89-1.53 (predicted)	DrugBank
Epirubicin	543.5193	-0.5	DrugBank
Vorinostat	264.3202	1.88-2 (predicted)	DrugBank
Alamar blue (resazurin)	229.037506	1.36	ChemSpider

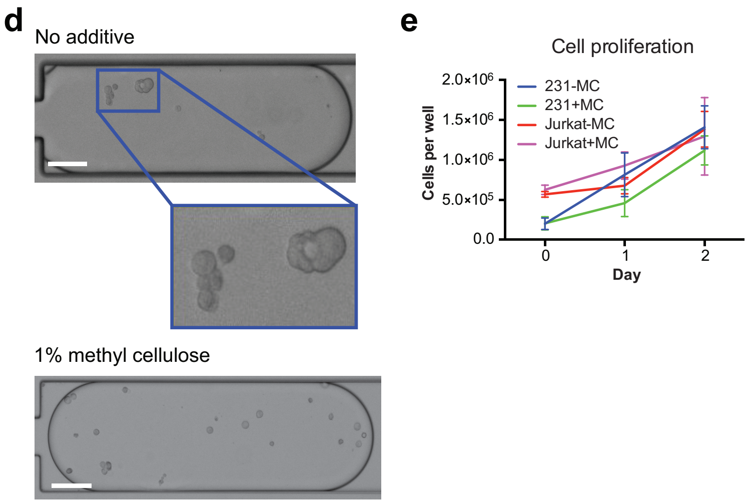

Supplementary Figure 3. Optimization of oil phase and aqueous phase of our microfluidic chip assay was performed. (a) Addition of 2% 008-Fluorosurfactant (Ran Biotechnologies, USA) in Fluorinert® FC-40 oil (Sigma-Aldrich, USA) prevented droplets in contact from coalescence and cross-contamination. No red fluorescence was observed in the right droplet even when it touched the left droplet containing strong red

fluorescence under illumination at 80% light intensity (Life Technologies EVOS FL Imaging System, USA), indicating that no cross-contamination between droplets occurred between droplets in contact. (b) Fluorosurfactant-emulsified droplets exhibited no fluorescent dye adsorption on PDMS walls of the channel after 15 h incubation at 37°C. Droplets containing red fluorescence dye were incubated at 37°C for 15 h, but no trace of fluorescent dye was observed along the PDMS walls as the droplet shrank due to evaporation. (c) Phase separation experiment using Alamar Blue dye in phosphate buffered saline (PBS) as aqueous phase and Fluorinert® FC-40 oil as oil phase with and without fluorosurfactant (Detergent) addition was performed in a CO_2 incubator (Static) or in a bacterial shaker with 200rpm shaking (Shake). Results showed that Tube 1 (Static + Detergent) that mostly mimicked droplet state on chip prevailed clean phase separation after overnight incubation, whereas shaking enhanced dye migration into the oil phase in Tube 2 (Shake + Detergent) and to a greater extent in absence of fluorosurfactant in Tube 4 (Shake - Detergent). Comparison of the formula weight and logP values of the drugs used in this assay and those of Alamar blue dye suggested that the observation from this experiment was likely to be relevant to the drugs. (d) Cell aggregation was abolished after adding methyl cellulose to culture medium to yield a final concentration of 1% (w/v). MDA-MB-231 cells formed aggregates without additive (left panel), whereas cells were suspended as single cells after overnight incubation in the presence of 1% (w/v) methyl cellulose (right panel). Scale bars indicated 25 μm. (e) Cell proliferation was measured by cell counting in the presence and absence of methyl cellulose. MDA-MB-231 cells and Jurkat cells were cultured on a 96-well plate at 37°C in a CO_2 incubator for 2 d in the presence and absence of 1% methyl cellulose (w/v). Cells were counted by cell counter (Nexcelom Cellometer Auto 2000, USA) using SD100 counting slides (Nexcelom, USA). Graph was plotted by the mean number of cells per well (y-axis) against the number of cultured days (x-axis); error bars denoted standard deviation of mean cell number per well from three replicates in parallel.

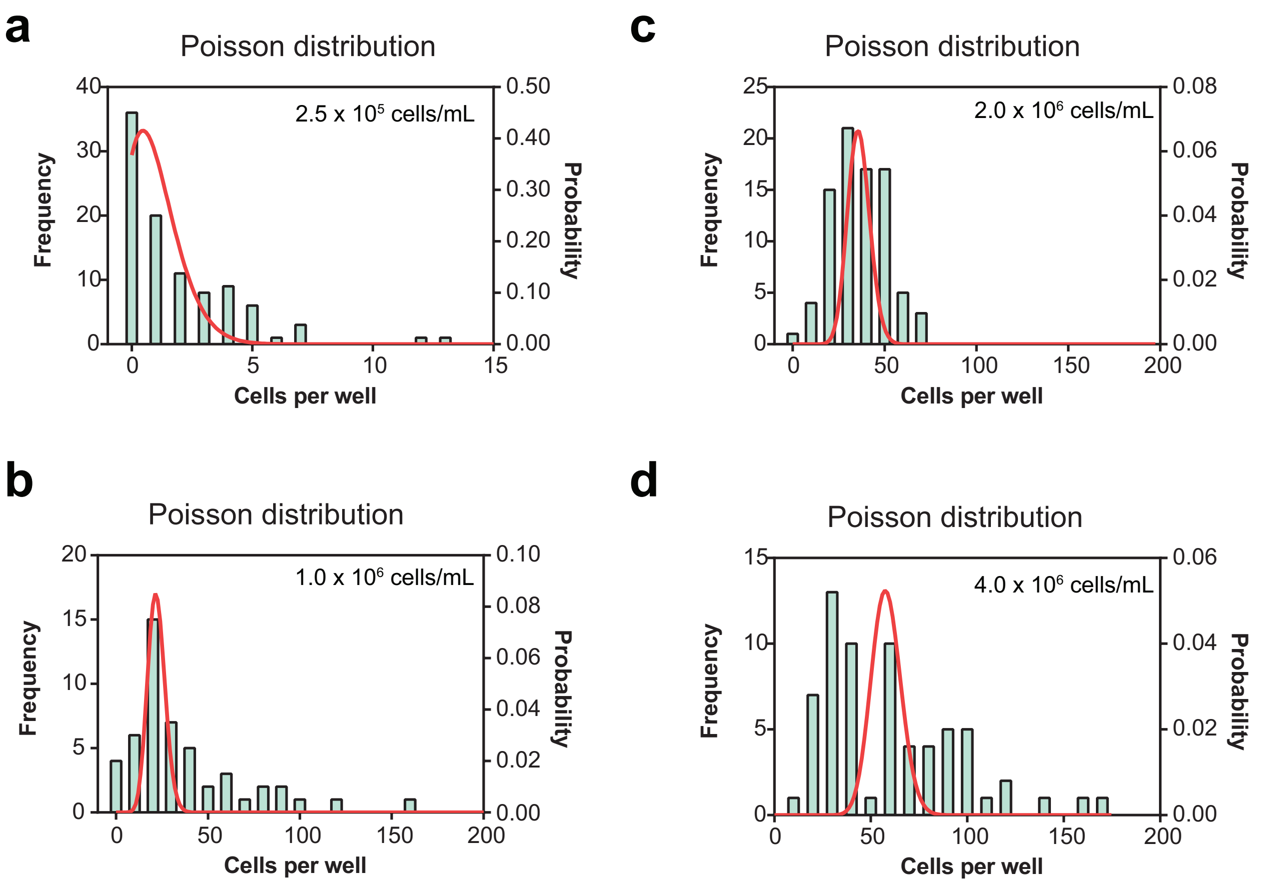

Supplementary Figure 4. Frequency plot of the total number of cells observed in each well prevailed good Poisson distribution at different cell concentrations of (a) 2.5×10^5 cells/mL, (b) 1.0×10^6 cells/mL, and (c) 2.0×10^6 cells/mL, while less optimal distribution due to minor cell aggregation was observed at 4.0×10^6 cells/mL. MDA-MB-231 cells were used for all experiments except that Jurkat cells were used for the concentration of 4.0×10^6 cells/mL.

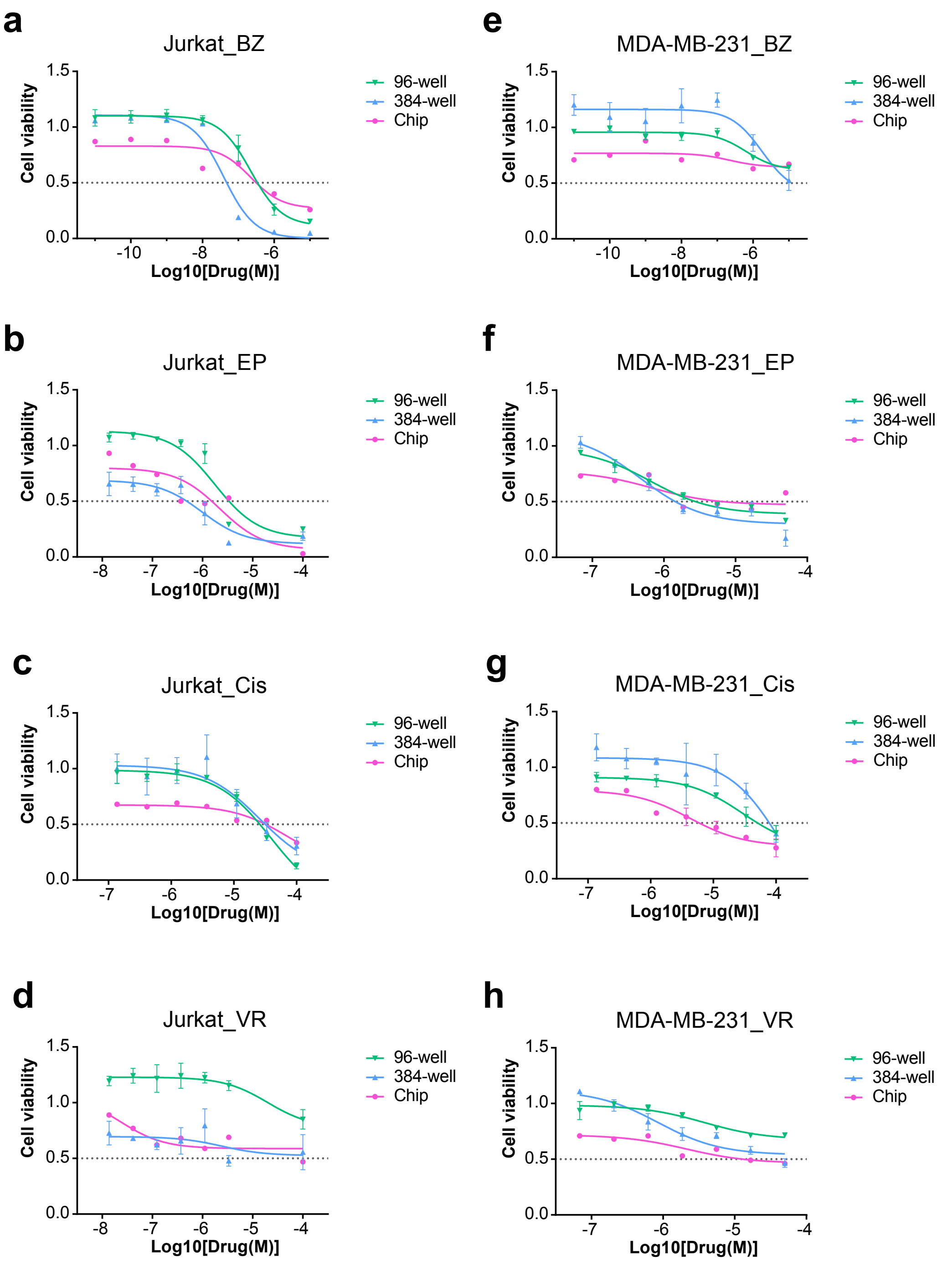

Supplementary Figure 5. Jurkat cells was used as suspended cancer cell model and screened against four drugs, namely (a) Bortezomib (BZ), (b) Epirubicin (EP), (c) Cisplatin (Cis), and (d) Vorinostat (VR); MDA-MB-231 cells was used as adherent cancer cell model and screened against four drugs, namely (e) Bortezomib (BZ), (f) Epirubicin (EP), (g) Cisplatin (Cis), and (h) Vorinostat (VR). A horizontal dashed line

was drawn at 50% cell viability for comparison of IC_{50} between different screening methods. All graphs were plotted by cell viability (y-axis) against the log of final drug concentration (x-axis); error bars denoted standard deviation of mean cell viability obtained from all replicates in parallel experiments in plate reader assays, whereas for chip assay, error bars denoted standard deviation of mean cell viability obtained from two independent experiments.

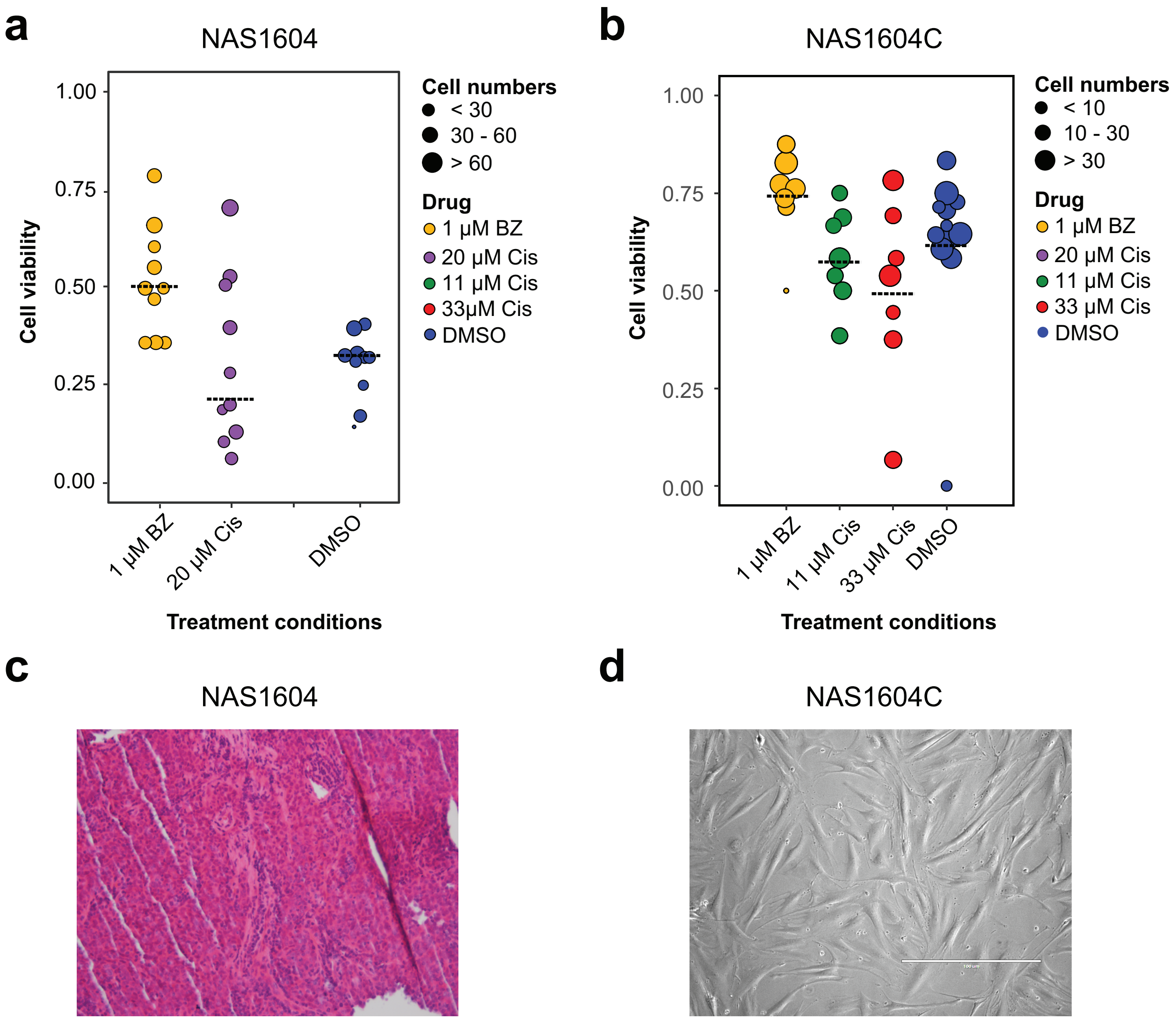

Supplementary Figure 6. Drug susceptibility between primary tumor and derived cell line of human nasopharyngeal tumor sample NAS1604 was shown. (a) Primary tumor dissociated cells of NAS1604 was screened against 1 µM Bortezomib and 20 µM Cisplatin (x-axis). (b) Primary tumor derived cell line NAS1604C was screened against 1 µM Bortezomib, 11 µM Cisplatin and 33 µM Cisplatin (x-axis). All graphs were plotted by cell viability (y-axis) against treatment conditions (x-axis); each dotted line indicated the mean cell viability of all droplets under the specified treatment conditions, whereas each dot indicated the mean cell viability of all cells in one droplet, and the size of the dot denoted the sample cell population size in one droplet. BZ denoted Bortezomib; Cis denoted Cisplatin. (c) Hematoxylin and Eosin stained tumor tissue section of primary nasopharyngeal tumor NAS1604 under 10x magnification depicted that the tumor was undifferentiated. (d) 10x magnified phase contrast image of the primary tumor derived cell line NAS1604C showed fibroblast-like cell morphology.

Supplementary Table 1. Drug list.

	Drug	Company	Cat#	Therapeutic target
1	Bortezomib<br>(Velcade)	BOC Sciences	179324-69-7	Proteasome inhibitor
2	Cisplatin<br>(*cis*-Diammineplatinum(II) dichloride)	Sigma-Aldrich	P4394	DNA crosslinking agent
3	Epirubicin hydrochloride	J&K	194237	Topoisomerase inhibitor
4	Vorinostat<br>(SAHA)	Selleckchem	S1047	HDAC inhibitor

Supplementary Table 2. Statistical analysis of drug response of NAS1608 human primary nasopharyngeal tumor sample towards Bortezomib and Cisplatin.

Statistical analysis of all five data sets of NAS1608 human primary nasopharyngeal tumor sample treated with 10 μM Bortezomib (BZ10), 1 μM Bortezomib (BZ1), 100 μM Cisplatin (Cis100), 33.3 μM Cisplatin (Cis33) and DMSO (DMSO) at 0 h and 17 h post-treatment was performed. First, two-way ANOVA was performed in Microsoft Excel. Next, post hoc analysis using Tukey's honest significant difference (HSD) test was performed in R v.3.3.2. Significant difference was highlighted in yellow.

Two-way ANOVA without replicates results

0 h treatment

Source	SS	DF	MS	F	p-value	Critical F
Row	0.265182	9	0.029465	1.510195	0.181749	2.152607
Column	0.066994	4	0.016749	0.858438	0.498091	2.633532
Error	0.702378	36	0.01951			
Total	1.034554	49				

17 h treatment

Source	SS	DF	MS	F	p-value	Critical F
Row	0.466516	9	0.051835	1.694114	0.126515	2.152607
Column	0.333928	4	0.083482	2.728423	0.044157	2.633532
Error	1.101499	36	0.030597			
Total	1.901943	49				

* Each set of treatment data was arranged as droplet cell viability in columns.

Tukey's HSD test results

Group	Mean difference	Lower boundary	Upper boundary	p-value
BZ10-BZ1	-0.07893	-0.26331	0.105445	0.739884
Cis100-BZ1	0.044038	-0.14034	0.228418	0.959567
Cis33-BZ1	-0.00306	-0.18744	0.181323	0.999999
DMSO-BZ1	0.138241	-0.06494	0.341418	0.313332
Cis100-BZ10	0.122973	-0.06141	0.307352	0.332825
Cis33-BZ10	0.075878	-0.1085	0.260258	0.766589
DMSO-BZ10	0.217176	0.013999	0.420353	0.030927
Cis33-Cis100	-0.04709	-0.23147	0.137285	0.948795
DMSO-Cis100	0.094203	-0.10897	0.29738	0.679704
DMSO-Cis33	0.141298	-0.06188	0.344475	0.292341

Supplementary Movie 1. Two color dyes were consecutively loaded in one channel as proof of concept of screening different conditions on chip. In this experiment, the food dyes were dissolved in water, representing the aqueous phase; the oil phase contained Fluorinert® FC-40 oil (Sigma-Aldrich, USA) supplemented with 2% 008-Fluorosurfactant (Ran Biotechnologies, USA). The sample loading workflow was illustrated. Firstly, we loaded the chip with oil. Next, food dyes of blue and green colors were segregated by translucent oil phase and loaded consecutively on chip. Each dye formed droplets in sequential order that was identical to the loading sequence.

Supplementary Movie 2. Microfluidic channels with different width ratios between the bypass channel and the neck were tested to achieve optimal well loading and droplet formation.
